# Association between Spirituality and Adherence to Management in Outpatients with Heart Failure

**DOI:** 10.5935/abc.20160076

**Published:** 2016-06

**Authors:** Juglans Souto Alvarez, Livia Adams Goldraich, Alice Hoefel Nunes, Mônica Cristina Brugalli Zandavalli, Rafaela Brugalli Zandavalli, Karlyse Claudino Belli, Neusa Sica da Rocha, Marcelo Pio de Almeida Fleck, Nadine Clausell

**Affiliations:** 1 Heart Failure Program, Division of Cardiology, Hospital de Clinicas de Porto Alegre, Universidade Federal do Rio Grande do Sul, Porto Alegre - Brazil; 2 Universidade Federal do Rio Grande do Sul, Porto Alegre - Brazil; 3 Hospital de Clinicas de Porto Alegre, Porto Alegre - Brazil; 4 Division of Psychiatry, Hospital de Clinicas de Porto Alegre, Universidade Federal do Rio Grande do Sul, Porto Alegre - Brazil

**Keywords:** Heart Failure, Medication Adherence / psychology, Surveys and Questionnaires, Outpatients, Spirituality, Religion

## Abstract

**Background:**

Spirituality may influence how patients cope with their illness.

**Objectives:**

We assessed whether spirituality may influence adherence to management of outpatients with heart failure.

**Methods:**

Cross sectional study enrolling consecutive ambulatory heart failure patients in whom adherence to multidisciplinary treatment was evaluated. Patients were assessed for quality of life, depression, religiosity and spirituality utilizing validated questionnaires. Correlations between adherence and psychosocial variables of interest were obtained. Logistic regression models explored independent predictors of adherence.

**Results:**

One hundred and thirty patients (age 60 ± 13 years; 67% male) were interviewed. Adequate adherence score was observed in 38.5% of the patients. Neither depression nor religiosity was correlated to adherence, when assessed separately. Interestingly, spirituality, when assessed by both total score sum (r = 0.26; p = 0.003) and by all specific domains, was positively correlated to adherence. Finally, the combination of spirituality, religiosity and personal beliefs was an independent predictor of adherence when adjusted for demographics, clinical characteristics and psychosocial instruments.

**Conclusion:**

Spirituality, religiosity and personal beliefs were the only variables consistently associated with compliance to medication in a cohort of outpatients with heart failure. Our data suggest that adequately addressing these aspects on patient’s care may lead to an improvement in adherence patterns in the complex heart failure management.

## Introduction

Heart failure (HF) continues to challenge multidisciplinary health care teams.^1^ Its prevalence remains elevated and its management usually requires poly-pharmacy along with satisfactory self-awareness of the disease.^2,3^ The course of HF, in its chronicity and frequently inexorable outcomes, shares similarities with many cancer diseases and resembles their impact on poor quality of life standards.^4^ Patients face important limitations to adequately adhere to the complexity of HF management.^5^

Adherence appears as an important aspect in the course of HF. It influences patients' pattern of decompensation and subsequent hospital re-admissions.^6^ In addition, adequate adherence standards may help to improve quality of life.^7^ Many factors are thought to influence patient adherence to HF management. Clinical aspects such as comorbidities commonly associated with HF have been addressed in this regard.^5^ Socio-economic background, psychological aspects and patients' level of formal education have also been investigated as influential in adherence patterns in the HF population.^8,9^ Nonetheless, data addressing such issues on large cohorts being followed in specialized HF clinics remains scarce.

Spirituality has recently been studied in the setting of chronic diseases with poor quality of life and predictable ominous outcomes. Spiritual wellbeing refers to one's spirituality as "the state of affairs".^10^ This concept has been applied to unravel specifics on spiritual status in the palliative care setting, but very little data exists on spirituality associated to such a chronic and prevalent condition as HF. Spirituality has been shown to potentially influence how patients with HF cope with their syndrome, consequently having an impact on functional status, health status and quality of life.^11,12^ Recent data indicates that spirituality could favorably influence mortality in patients with HF.^13^ However, how spirituality relates to adherence patterns in stable outpatients with HF remains relatively unexplored. Few reports with controversial results, partially limited by convenience sampling and instruments utilized, failed to demonstrate a positive association between spirituality and degree of compliance in HF patients.^14^ Nevertheless, for many other chronic disorders there is sustained evidence that spirituality could improve compliance.^15^

In this study, we examined associations between spirituality and adherence to management in outpatients with HF, independently of psychosocial and educational background.

### Patients and Methods

#### Study design

This is a cross-sectional study which enrolled HF outpatients followed at a tertiary care University Hospital in Porto Alegre, Brazil, from August, 2012 to June, 2013. The study protocol was approved by the Institutional Research and Ethics Committee and all enrolled participants signed a written informed consent prior to study entry.

#### Participants

Consecutive ambulatory patients (mainly composed by patients with newly diagnosed left ventricular dysfunction, post HF hospital admission and/or refractory symptoms) being followed for a minimum of six months in the HF Clinic at the Hospital de Clínicas de Porto Alegre were invited to participate. Patients in any New York Heart Association functional class, regardless of HF etiology, were eligible. Exclusion criteria were inability to understand the study protocol and to answer the questions without assistance due to cognitive impairment or auditory deficit.

#### Study end-points and procedures

Patients were assessed for adherence to therapy, quality of life, depression, religiosity and spirituality utilizing validated questionnaires. All utilized instruments were previously validated to Brazilian Portuguese language.^16-21^ Interviews were performed following the clinic appointment by research staff previously trained in questionnaire application. Time required for answering all the instruments ranged from 50 to 70 minutes. Patients answered questions orally and staff filled questionnaires as requested. Demographics and clinical characteristics were obtained from electronic chart review and clinical data were acquired during the clinic visit by a researcher who was unaware of questionnaire results. Definitions of psychosocial variables of interest are detailed below.

***Adherence to therapy.*** The adherence to pharmacologic and non-pharmacologic therapy was assessed according to the Repetitive Education and Monitoring For Adherence for Heart Failure (REMADHE) study protocol, which has been adapted and is currently used in clinical practice in our HF Clinic.^17,22^ The questionnaire is composed by ten-questions involving four domains: use of medications (one question); food and fluids (seven questions); alcohol consumption (one question); and medical appointments (one question). The score ranges between 0 and 26 points, with higher scores indicating better patient's adherence. A REMADHE score equal to or higher than 18 points indicates adequate level of adherence.^22^

**Quality of life:** Two instruments were used to assess quality of life: generic and disease-specific questionnaires. Generic quality of life assessment was performed with the utilization of the World Health Organization Quality of Life (WHOQoL-Bref) while disease-specific was assessed by the Minnesota Living With Heart Failure Questionnaire (MLHFQ).^23,24^ The WHOQoL-Bref is an abbreviated version of the WHOQOL-100 which is composed of 26 questions: a question about quality of life in general, a question about satisfaction with one's self health status, and 24 questions divided into four domains - physical, psychological, social relations and environment. The MLHFQ evaluates quality of life related to HF symptomatology within the previous month and correlates proportionally to functional class.^25^ Higher WHOQoL-Bref scores indicate better quality of life in general, whereas lower MLHFQ represent better HF-related quality of life.

**Depression:** Depression was evaluated by the Patient Health Questionnaire (PHQ-9), which is a screening tool for detection of depression, based on symptom occurrence within the previous two weeks. It comprehends nine questions based on the major criteria for the diagnosis of major depression according to the Diagnostic and Statistical Manual of Mental Disorders, 4th edition (DSM-IV).^26,27^ Depression is classified, according to the score, as moderate depressive symptoms (total score between 10 and 14), moderate major depression (score between 15 and 19) and severe major depression (score equal or higher than 20).^26^

**Religiosity, Spirituality and Personal Beliefs:** Two instruments were used to evaluate these dimensions.

1. ) The Duke University Religion Index (DUREL) scale is a tool for assessment of spirituality that is focused on religious aspects.^28^ Its transcultural adaptation was developed and validated by Moreira-Almeida.^18^ The DUREL scale has five items that describe three dimensions of religiosity, known to best correlate with health-related outcomes: organizational (ORA); non-organizational (NORA); and intrinsic religiosity (IR). The score ranges from 1 to 30 points and higher scores indicate elevated levels of religiosity.
2. ) The World Health Organization Quality of Life Spirituality, Religiosity and Personal Beliefs (WHOQoL-SRPB) instrument is an additional module of the WHOQOL to evaluate spirituality, religion and personal beliefs (SRPB) as a component of the quality of life construct. It is composed by 32 items distributed in eight factors (Spiritual Connection, Meaning of Life, Awe & Wonder, Wholeness & Integration, Spiritual Strength, Inner Peace, Hope & Optimism and Faith) in a general index composed of 4 items (SRPB Global), originally of the SRPB domain of the WHOQOL-100.^19,29^

#### Statistical analyses

Normally distributed (according to Shapiro-Wilks testing) continuous variables were expressed as mean ± standard deviation, while non-normally distributed ones were expressed as median and interquartile ranges. Categorical variables were reported as absolute numbers and percentages. Normally distributed continuous variables were analyzed by unpaired *t-*test. Non-normally distributed continuous variables were analyzed using Mann Whitney U test. Chi-square test (or exact Fisher test when appropriate) was used to compare categorical variables. Spearman coefficients were used for evaluation of correlations between adherence and psychosocial variables of interest. Kruskal Wallis was used to compare scores of spirituality across REMADHE quartiles. Logistic regression models were used to explore the association of spirituality to an adequate level of adherence (REMADHE ≥ 18 points). Adjusting covariates for multivariable models were tested for colinearity and selected among demographic, clinical and psychosocial variables of either clinical or statistical significance. The report by Black and co-workers, that correlated spirituality and adherence utilizing different instruments, was used to estimate a sample size of 130 subjects in the current study (α = 5%, β = 80%; effect size 25%).^14^ All analyses were performed using the SPSS 20.0 statistical package (SPSS Inc., Chicago, IL, USA). A p value lower than 0.05 was considered of statistical significance.

## Results

One hundred and thirty patients were interviewed between August, 2012 and June, 2013. Demographic and clinical characteristics of the studied population are detailed in Table 1. A description of the average scores obtained through the study instruments is provided in Table 2. Overall, there was a low level of adherence, according to REMADHE scores. Adequate adherence was observed in 38.5% of the population.

**Table 1:** Demographics and clinical characteristics of the study population

N		130
**Demographics**		
**Age, years**		60 ± 13
**Gender, male**		88 (67.5%)
**Ethnicity**		
	Caucasian	113 (87%)
	African-descendent	9 (7%)
	Other	8 (6%)
**Education**		
	Functionally illiterate	5 (4%)
	Elementary, non-graduated	79 (61%)
	Elementary, graduated	26 (20%)
	High school, non-graduated	6 (5%)
	High school, graduated	14 (11%)
**Marital status**		
	Single	31 (24%)
	Married	78 (60%)
	Divorced	15 (11.5%)
	Widowed	6 (4.5%)
Heart failure history		
**Etiology**		
	Ischemic	42 (32.5%)
	Idiopathic	28 (21.5%)
	Hypertensive	29 (22.5%)
	Valvular	14 (10%)
	Alcoholic	10 (7.5%)
	Other	7 (5%)
Ejection fraction, %		36 ± 13
**Functional class, NYHA**		
	I-II	97 (74.5%)
	III-IV	33 (25.5%)
Cardiac devices (ICD or CRT-D)		23 (18%)
**Hospital admissions in the previous year**		
	None	82 (63%)
	One	26 (20%)
	More than one	22 (17%)
**Comorbidities**		
	Hypertension	75 (57.5%)
	Dyslipidemia	64 (49%)
	Previous myocardial infarction	39 (30%)
	Previous cardiac surgery	24 (18.5%)
	Diabetes	50 (38.5%)
	COPD	10 (7.5%)
	Chronic kidney impairment	52 (40%)
	Previous stroke	20 (15.5%)
	Collagen tissue disease	11 (9%)
	Smoking, past or present	66 (50.5%)
	Alcoholism, past or present	29 (22.5%)
	Neoplasia	18 (14%)

NYHA: New York Heart Association; ICD: implantable cardiac defibrillator; CRT-D: cardiac resynchronization therapy-defibrillator; COPD: chronic obstructive pulmonary disease. Data expressed as mean ± SD and number (percentage). Ejection fraction assessed by bi-dimensional echocardiography (Simpson method).

**Table 2:** Description of psychosocial instruments applied in the study population

N		130
**Adherence (REMADHE)**		16.2 ± 4.1
**Quality of life**		
**Generic (WHOQoL-Bref)**		
	Total	13.0 ± 3.7
	Domains	
	Physical	12.2 ± 3.1
	Psychological	14.1 ± 2.7
	Social	13.7 ± 2.1
	Environmental	14.8 ± 1.9
Disease-specific (MLHFQ)		50.5 ± 16.9
**Depression (PHQ-9)**		4.8 ± 5.3
**Religiosity (DUREL)**		
Total		23.5 ± 4.6
	Intrinsic	15.5 ± 2.8
	Organizational	3.3 ± 1.6
	Non-organizational	4.7 ± 1.3
**Spirituality (WHOQoL-SRPB)**		
Total		3.8 ± 0.61
Domains		
	Connect	3.7 ± 0.7
	Meaning	3.9 ± 0.6
	Awe	3.8 ± 0.8
	Whole	3.7 ± 0.6
	Strength	3.8 ± 0.8
	Peace	3.8 ± 0.7
	Hope	3.8 ± 0.8
	Faith	3.8 ± 0.7

REMADHE: Repetitive Education and Monitoring for Adherence for Heart Failure; WHOQoL-Bref: World Health Organization Quality of Life; MLHFQ: Minnesota Living with Heart Failure Questionnaire; PHQ-9: Patient Health Questionnaire 9; DUREL: Duke University Religion Index; WHOQoL-SRPB: World Health Organization Quality of Life Spirituality, Religiosity and Personal Beliefs; Data expressed as mean ± SD.

**Figure 1:**
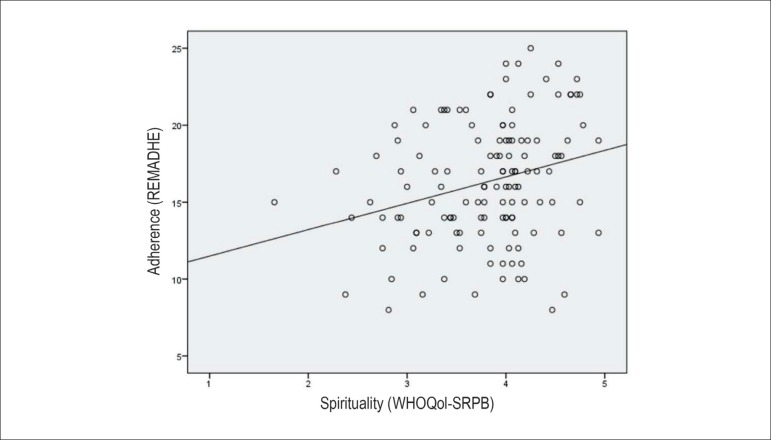
Correlation plot between adherence (REMADHE) and spirituality (WHOQoL-SRPB) scores (Spearman coefficient = 0.26; p = 0.003)

**Figure 2:**
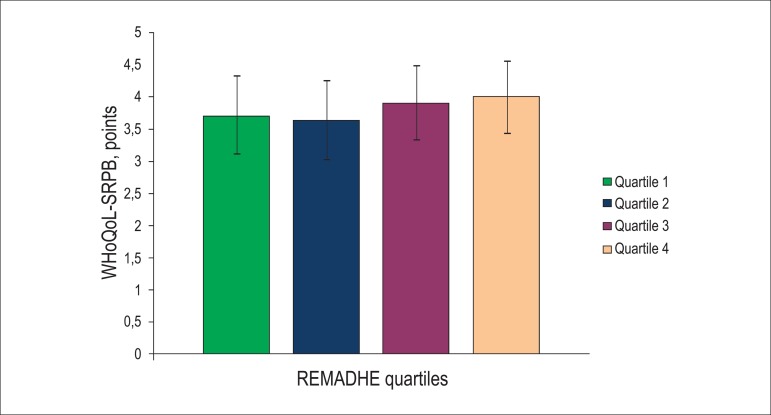
Score of spirituality (WHOQoL-SRPB) according to quartiles of adherence as per REMADHE score. p-value of 0.052 by Kruskal-Wallis test Error bars represent standard-deviation.

The correlations between the adherence score with clinical characteristics and psychosocial scores are demonstrated in Table 3. A description of associations of demographic and clinical variables with the adherence score is also described in the Supplemental Table. Among demographics, REMADHE score differed only according to marital status. Clinical characteristics associated to higher adherence scores were ischemic HF etiology, presence of an implantable cardiac defibrillator and chronic kidney impairment. Adherence was positively correlated to the generic quality-of-life measure, but not to the disease-specific HF score. Neither depression nor religiosity was correlated to adherence. Interestingly, spirituality, when assessed by both total WHOQoL-SRPB score sum and by many specific domains, was positively correlated to adherence. Although significantly correlated to adherence score, the magnitude of spirituality association was relatively weak (Figure 1). Notably, there was a trend towards higher spirituality scores across quartiles of the adherence score (Figure 2).

**Table 3:** Correlations of clinical and psychosocial variables with adherence score (REMADHE)

		r	p
**Clinical characteristics**			
	Age, years	0.10	0.24
	Ejection fraction, %	-0.09	0.30
	**NYHA functional class**	0.03	0.70
**Quality of Life**			
**Generic (WHOQoL-Bref)**			
Total		0.21	0.02
Domains			
	Physical	0.13	0.16
	Psychological	0.28	0.001
	Social	0.08	0.36
	Environmental	0.21	0.01
**Disease-specific (MLHFQ)**		-0.09	0.29
**Depression (PHQ-9)**		-0.12	0.16
**Religiosity (DUREL)**			
**Total**		0.13	0.14
	Intrinsic	0.20	0.02
	Organizational	0.02	0.79
	Non-organizational	-0.006	0.95
**Spirituality (WHOQoL-SRPB)**			
Total		0.26	0.003
Domains			
	Connect	0.31	< 0.0001
	Meaning	0.23	0.008
	Awe	0.27	0.002
	Whole	0.19	0.02
	Strength	0.21	0.02
	Peace	0.23	0.01
	Hope	0.19	0.03
	Faith	0.27	0.002

REMADHE: Repetitive Education and Monitoring for Adherence for Heart Failure; NYHA: New York Heart Association; WHOQoL-Bref: World Health Organization Quality of Life; MLHFQ: Minnesota Living with Heart Failure Questionnaire; PHQ-9: Patient Health Questionnaire 9; DUREL: Duke University Religion Index; WHOQoL-SRPB: World Health Organization Quality of Life Spirituality, Religiosity and Personal Beliefs; r: indicates Spearman coefficients; p: for Spearman coefficients.

Spirituality was also found to be significantly correlated to other psychosocial variables evaluated. WHOQoL-SRPB was moderately correlated to both generic (WHOQoL-Bref [r = 0.47; p = 0.0001]) and disease-specific (MLHFQ [r = -0.34; p = 0.0001]) measures of quality-of-life. There was an inverse correlation between WHOQoL-SRPB and depression classification by PHQ-9 (r = -0.49; p < 0.0001). Of note, there was a positive correlation between WHOQoL-SRPB and religiosity assessed by DUREL (r= 0.55; p= 0.0001), which was also observed within domains of both instruments (Table 4).

**Table 4:** Correlations between spirituality and religiosity scores

		DUREL		DUREL-ORA		DUREL-NORA		DUREL-IR	
		r	p	r	p	r	p	r	p
**WHOQoL-SRPB**		0.55	0.0001	0.36	0.0001	0.29	0.001	0.57	0.0001
	Connect	0.54	0.0001	0.29	0.0001	0.34	0.0001	0.59	0.0001
	Meaning	0.38	0.0001	0.19	0.03	0.18	0.04	0.44	0.0001
	Awe	0.36	0.0001	0.27	0.02	0.10	0.25	0.38	0.0001
	Whole	0.47	0.0001	0.30	0.0001	0.27	0.002	0.48	0.0001
	Strength	0.49	0.0001	0.33	0.0001	0.28	0.001	0.51	0.0001
	Peace	0.39	0.0001	0.27	0.002	0.20	0.02	0.41	0.0001
	Hope	0.37	0.0001	0.29	0.001	0.22	0.01	0.31	0.0001
	Faith	0.54	0.0001	0.33	0.0001	0.34	0.0001	0.56	0.0001

DUREL: Duke University Religion Index; ORA: organizational religious activity; NORA: non-organizational religious activity; IR: intrinsic religiosity; WHOQoL-SRPB: World Health Organization Quality of Life Spirituality, Religiosity and Personal Beliefs; r: indicates Spearman coefficient; p: for Spearman coefficients.

Among multivariable models to identify clinical and psychosocial variables associated to the presence of adequate adherence, WHOQoL-SRPB was an independent predictor when adjusted for demographics, clinical characteristics and psychosocial instruments (Table 5). Aside from WHOQoL-SRBP, DUREL was the only additional psychosocial instrument to demonstrate borderline significance for association to adequate adherence.

**Table 5:** Logistic regression models for association of spirituality (WHOQoL-SRPB) to adequate adherence to therapy (REMADHE ≥ 18 points)

**Model 1**	**β coefficient**	**OR (CI 95%)**	**p**
WHOQoL-SRPB, 1-point increase	1.01	2.76 (1.31 – 5.81)	0.007
Age, 1-year increase	-0.01	0.98 (0.95 – 1.01)	0.32
Ejection fraction, 1% increase	-0.01	0.98 (0.95 – 1.02)	0.40
Marital status, married	0.56	1.75 (0.76 – 4.08)	0.19
Instruction, ≥ elementary school graduation	0.31	1.36 (0.59 – 3.11)	0.47
**Model 2**	**β coefficient**	**OR (CI 95%)**	**p**
WHOQoL-SRPB, 1-point increase	1.17	3.23 (1.49 – 7.01)	0.003
Heart failure of ischemic etiology	-0.31	0.73 (0.32 – 1.67)	0.45
Implantable cardiac defibrillator	-0.91	0.40 (0.15 – 1.05)	0.06
Chronic kidney disease	-0.72	0.48 (0.21 – 1.08)	0.08
Marital status, married	-0.36	0.69 (0.31 – 1.57)	0.38
**Model 3**	**β coefficient**	**OR (CI 95%)**	**p**
WHOQoL-SRPB	-0.12	4.89 (1.64 – 14.58)	0.004
WHOQoL-Bref	1.59	1.03 (0.98 – 1.06)	0.19
MLHFQ	0.03	1.02 (0.98 – 1.06)	0.26
PHQ-9	0.02	1.03 (0.92 – 1.16)	0.60
DUREL	0.03	0.89 (0.79 – 1.00)	0.05

WHOQoL-SRPB: World Health Organization Quality of Life Spirituality, Religiosity and Personal Beliefs; REMADHE: Repetitive Education and Monitoring for Adherence for Heart Failure; OR: odds ratio; CI: confidence interval; WHOQoL-Bref: World Health Organization Quality of Life; MLHFQ: Minnesota Living with Heart Failure Questionnaire; PHQ-9: Patient Health Questionnaire 9; DUREL: Duke University Religion Index.

Model 1 – adjusted for demographic and clinical variables selected by clinical significance;

Model 2 – adjusted for demographic and clinical variables selected by significance in univariate analyses;

Model 3 – adjusted for other psychosocial instruments of quality-of-life, depression and religiosity; Odds ratio represents the magnitude of association per 1-point increase in each score

## Discussion

The main finding of the present study is that SRPB were consistently associated with adherence to treatment in a cohort of HF patients followed in a tertiary care clinic. Importantly, in our study, this association was independent of relevant demographic and clinical data known to influence adherence to HF management.

This is the first study to show a clear association of spirituality and adherence to treatment in HF. However, our study cannot determine if there is a direct effect of spirituality in adherence or if spirituality is only a marker of broader and more complex effect. For example, someone who is spiritualized is probably more prone to follow recommendations coming from someone he has a close relationship with (e.g., a physician). We identified three previous studies addressing the possible interactions between HF and spirituality.^14,30,31^ Black et al.^14^ sent a package of instruments (Spiritual Assessment Scale and the Heart Compliance Questionnaire) by mail to a convenience sample of 213 patients with a return rate of 45%. The authors did not find a significant correlation between spirituality and compliance. The study by Thomas^30^ using a convenience sample of 97 patients showed a positive result with moral-ethical-spiritual self which accounted for 10.8% of the variance in adherence. Dickson et al.^31^ studying socio-cultural influences on HF self-care in an ethnic minority, black population, using a mixed-methods strategy, found that spirituality was linked to self-care. More recently, issues related to wellbeing were shown to impact positively in patients with stage B asymptomatic HF - spirituality apparently played a role in mediating these effects.^32^

Although religiosity and spirituality have been associated with better healthcare practices, such observations failed to translate into better cardiovascular disease outcome in an adequately-powered study.^33-35^ The hypothesis that SRPB could affect compliance in chronic diseases and, particularly, in HF has also been raised by different authors.^14,30,36^ There are some possible models proposed to explain such relationship. Black et al.^14^ suggested that spiritual beliefs influence health beliefs which could lead to the practice of health-related activities such as the use of medications, control of weight, and diet compliance. Thomas^30^ applied the Roy's Self Concept model to identify several potential predictors of medical compliance.^30,7^ In this model, any stimulus is perceived either as a threat or a challenge to one's self-concept of body image, body sensation, self-consistency and moral-ethical-spiritual self. Briefly, stimuli perceived as a threat are reacted to in a negative way and consequently avoided, while stimuli perceived as a challenge are reacted to in a positive way and consequently followed. Thomas^30^ found that patients who perceived the HF regimen as a threat either to body image, self-consistency, body sensation or self-ideal were less likely to adhere to it. On the contrary, those to whom the regimen was perceived as a challenge to moral-ethical-spiritual self were most likely to adhere to medical therapy. Lastly, a recent survey conducted on HF patients showed that they would have welcomed spiritual care in their management.^38^

Our study has some limitations. First, as we used a cross-sectional design we can only conclude about association between spirituality and adherence, but not a causal relationship. Second, our sample was obtained in Brazil, a country where spirituality and religion are very notoriously important values. Additional studies are necessary to assess if these findings are replicable in different cultural and religious backgrounds. Finally, the effect of spirituality in adherence to different aspects of the HF management - pharmacological and non-pharmacological therapy - was not individually assessed. The REMADHE tool used in our study does not discriminate between the various components of the HF management in depth. If available, such information could be useful to better allocate the role of multidisciplinary care, *vis a vis* the spirituality of patients, and be taken into consideration accordingly, to improve patients' adherence.

## Conclusions

Our study highlights that spirituality could be an important variable associated with adherence to treatment in the setting of outpatients with HF, suggesting that physicians and health professionals should be aware of its importance to improve clinical practice outcomes and implement measures to address the spiritual needs of patients. Further studies are warranted to better determine whether pharmacologic and non-pharmacologic measures in the management of HF are equally influenced by spirituality-related behavior.

## Supplemental Table

**Table:** Adherence score according to demographics and clinical characteristics.

		REMADHE, points	p (t test)
**Demographics**			
**Age**			
	< 60	15.9 ± 4.1	0.31
	≥ 60	16.6 ± 3.7	
**Gender**			
	Male	16.6 ± 3.8	0.16
	Female	15.6 ± 4.2	
**Ethnicity**			
	Caucasian	16.5 ± 3.9	0.23
	Other	15.2 ± 3.8	
**Education**			
	Elementary graduated or higher degree	16.0 ± 3.8	0.56
	Other	16.5 ± 4.0	
**Marital status**			
	Married	17.0 ± 4.1	0.01
	Other	15.2 ± 3.5	
**Heart failure history**			
**Etiology**			
	Ischemic	17.4 ± 3.6	0.02
	Non-ischemic	15.8 ± 4.0	
**Ejection fraction**			
	≤ 35%	16.4 ± 3.9	0.83
	> 35%	16.2 ± 3.9	
**Functional class, NYHA**			
	I-II	16.1 ± 4.0	0.38
	III-IV	16.8 ± 3.7	
**Cardiac Defibrillator**			
	Yes	17.7± 3.2	0.05
	No	16.0 ± 4.1	
**Admissions in the previous year**			
	None	16.1 ± 4.0	0.52
	Any	16.6 ± 3.8	
**Comorbidities**			
**Hypertension**			
	Yes	16.6 ± 3.7	0.34
	No	15.9 ± 4.2	
**Previous myocardial infarction**			
	Yes	17.2 ± 3.6	0.11
	No	15.9 ± 4.0	
**Previous cardiac surgery**			
	Yes	16.5 ± 4.4	0.8
	No	16.3 ± 3.9	
**Diabetes**			
	Yes	16.0 ± 3.7	0.50
	No	16.5 ± 4.0	
**Chronic kidney impairment**			
	Yes	17.2 ± 3.8	0.04
	No	15.8 ± 3.9	
**Previous stroke**			
	Yes	16.6 ± 2.7	0.69
	No	16.3 ± 4.1	
**Smoking, past or present**			
	Yes	14.3 ± 4.1	0.28
	No	15.8 ± 4.2	
**Alcoholism, past or present**			
	Yes	16.1 ± 4.0	0.34
	No	16.9 ± 3.7	
**Neoplasia**			
	Yes	15.6 ± 4.4	0.38
	No	16.4 ± 3.9	
